# To Prevent Oxidative Stress, What about Protoporphyrin IX, Biliverdin, and Bilirubin?

**DOI:** 10.3390/antiox12091662

**Published:** 2023-08-23

**Authors:** Ana Martínez, Isabel López-Rull, Juan A. Fargallo

**Affiliations:** 1 Departamento de Materiales de Baja Dimensionalidad, Instituto de Investigaciones en Materiales, Universidad Nacional Autónoma de México, Circuito Exterior S. N., Ciudad Universitaria, Ciudad de México 04510, Mexico; 2 Departamento Biología y Geología, Física y Química Inorgánica, Área de Biodiversidad y Conservación, Universidad Rey Juan Carlos, C/Tulipán s/n., 28933 Madrid, Spain; isabel.lopez.rull@urjc.es; 3 Departamento de Ecología Evolutiva, Museo Nacional de Ciencias Naturales-CSIC, C/José Gutiérrez Abascal 2, 28006 Madrid, Spain; fargallo@mncn.csic.es

**Keywords:** oxidative stress, Donor Acceptor Map, photoactivation, microbial defense, sexual selection

## Abstract

The pigments responsible for eggshell color and patterning in birds are protoporphyrin IX (PP) and biliverdin (BV). Both are involved in the catalytic degradation of the hemo group. Bilirubin (BR), another pigment, is produced when BV is broken down. PP, BV, and BR are free radical scavengers. In this study, we theoretically investigated the antioxidant capacities of these three biological meaningful molecules using Density Functional Theory calculations. First, two antioxidant mechanisms were analyzed for PP, BV, and BR: electron transfer and Hydrogen Atom Transfer. Second, since PP and BV interact with the calcium carbonate matrix of the eggshell, we analyzed the interaction of these pigments with Ca^2+^ and investigated their chelate compounds. Third, we explored the pro-oxidant properties of PP and BV, which have been proposed for PP when photoactivated to the triplet state, but not for BV. Our results show that PP, BV, and BR are just as good antiradical as other important natural pigments (carotenoids). Neither the antiradical properties of PP and BV nor the UV-visible spectra change due to the presence of calcium, suggesting that the signaling function of these pigments is not affected by the link with Ca^2+^. Finally, we found that both PP and BV (alone and when linked to Ca^2+^) can transfer energy from its triplet state to molecular-oxygen-producing singlet oxygen, indicating their pro-oxidant capacity. This investigation answers important questions about the function of these pigments, which may help to understand their influence on the reproductive success of birds.

## 1. Introduction

Evolution has generated a wide range of color patterns in eggshells by combining two main pigments: protoporphyrin IX (PP) which produces reddish-brown colors, and biliverdin (BV) which produces blue and green [1,2,3,4,5]. Both pigments are similar in their tetrapyrrolic structure with PP-forming rings, whereas BV is an open chain [6]. PP occurs widely in biological systems, complexing with divalent cations such as Fe (haem), Mg (to form a major constituent of chlorophyll), and Zn (zinc protoporphyrin) [6]. BV is the metabolite produced by the catalytic degradation of the hemo group [7,8,9]. It breaks down into bilirubin (BR), giving rise to a yellowish color pigment. 

The synthesis of eggshell pigments occurs in the shell gland [10,11]. Subsequently, the pigments are deposited in the egg during shell formation [6,12] and are found throughout the egg white calcium carbonate matrix and cuticle [13]. The hypotheses that have been proposed to rationalize the adaptability of eggshell pigmentation has been divided into two possible explanations that are not mutually exclusive: one based on coloration as an attribute that is perceived by others, thus providing the functions of camouflage [14,15,16] or signaling [17]; and another attending to the physicochemical properties of the pigments and their effects on the embryo. Among the latter, PP improves thermoregulation, eggshell resistance, and antimicrobial defense through photoactivation [18,19,20,21,22,23]. For BV, benefits include the acceleration of embryonic development, protection against UV radiation, and prevention against oxidative stress [24,25]. It was also reported that the shell membrane has stronger antioxidant and antimicrobial properties when BV is present [25].

Although PP and BV are normally concentrated on the outer shell surface, there are many bird species that incorporate these pigments on the inner surface of the eggshell [25]. The hypothesis is that BV leaks from the inner surface of the eggshell into the egg, providing a benefit to the embryo through its ability to scavenge free radicals, reduce mutations, and inhibit viral replication. This protection is valuable because during the incubation period the embryo is under increasing oxidative stress [25]. It was also reported that BV neutralizes the oxidative action of pathogens that penetrate the eggshell and protects the shell membrane from oxidation, promoting the antioxidant and antimicrobial capacities of the eggshell membrane [25]. Recent evidence suggests that when BV passes from the outside to the inside of the eggshell, it can likewise protect the embryo through its conversion to BR, which is a molecule with high antioxidant capacity due to its reaction with superoxide anion [26,27,28]. It can also be hypothesized that the avian embryo absorbs small amounts of BV from the eggshell, as it does with calcium [25]. The antioxidant activity of BV and BR may be particularly important in the early stages of embryo development, when the levels of other natural antioxidants are low. 

As we already pointed out, BV has the ability to scavenge free radicals and reduce mutations [24,25,26,27,28]. BR is also considered a powerful antioxidant molecule. The extended system of conjugated double bonds and two reactive hydrogen atoms are those that are related to antioxidant activity via Hydrogen Atom Transfer (HAT) to an incipient radical [28]. In contrast, PP has been reported to be a pro-oxidant that induces oxidative stress and, as a consequence, causes liver damage [29]. PP is a pro-oxidant since it produces free radicals. The suggested mechanism is that the PP is photoactivated by sunlight and transformed to an excited triplet state. PP can transfer energy from its triplet state to molecular-oxygen-producing singlet oxygen (1O_2_), which is the main mediator of photochemical cell damage [22]. This production of free radicals is one of the explanations that has been reported for PP to describe the antimicrobial defense through photoactivation. It is important to note that PP is a pro-oxidant when it is in the excited (triplet) state, but it is not known whether PP in the ground state (singlet) is also a free radical scavenger.

**Figure 1:**
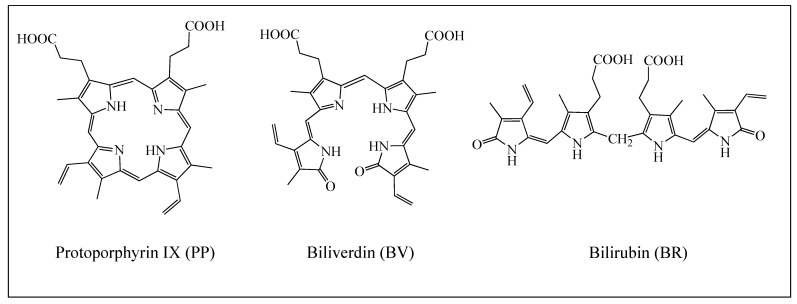
Schematic representation of BV, PP, and BR.

In spite of all the results reported until now, there are no theoretical studies concerning the antioxidant or pro-oxidant activity of PP, BV, and BR. The molecular structures of these three molecules are shown in Figure 1. In this investigation, we analyze and compare the antioxidant capacities of these three molecules, and we discover whether their possible mechanism of action is via electron transfer or HAT. We compare these properties with those of other important naturally occurring pigments, such as carotenoids [30,31,32,33,34]. Since PP and BV are found throughout the egg white calcium carbonate matrix, we analyze the interaction of these pigments with Ca^2+^. It is known that PP can be removed from freshly laid eggs [23] by rubbing their surface, but for nonrecently laid eggs, pigment removal with this procedure is not possible. The hypothesis that we propose to explain this observation is that PP forms a compound with Ca^2+^ and this incorporates the pigment into the calcium carbonate matrix. Once this occurs, PP cannot be removed from the eggshell. For BV, this was not observed, but we also analyzed the interaction with Ca^2+^ of this molecule to see the differences. We calculated UV-visible spectra to analyze color variations and investigated the antioxidant properties of these two calcium-containing pigments. In addition to the effect of Ca^2+^ on antioxidant properties, it is also important to investigate why PP is pro-oxidant and BV is not. To answer this question, we calculate the excited states of both pigments. This research answers important questions about the function of these pigments, which may help us understand their influence on avian reproductive success.

## 2. Materials and Methods

Gaussian09 was used for all electronic calculations [35]. Geometry optimizations of initial geometries were obtained at the M062x/LANL2DZ level of theory without symmetry constraints [36,37,38]. Harmonic analyses verified local minima. LANL2DZ is a pseudo-potential available for a variety of elements. These potentials have not been defined for elements H–Ne. For these elements, all-electron double zeta basis sets developed by Dunning (D95V) were used [39]. To compare the results obtained with different basis sets, the optimized geometry of BV was also obtained at the M062x/6-311+g(2d,p) level of theory. The cartesian coordinates of both optimized geometries are reported as Supplementary Material. Some results concerning the electron transfer properties were also obtained at both levels of theory, and the results are also included as Supplementary Material. As can be seen, both optimized structures and the results of the electron transfer process are similar. Therefore, we decided to continue at the M062x/LANL2DZ level of theory. 

The absorption spectra were computed with Time-Dependent Density Functional Theory (TDDFT) using single-point calculations of the optimized geometries, at the same level of theory in benzene and water, to mimic nonpolar and polar environments [40,41,42]. To obtain the excitation energies, single-point calculations of singlets and triplets were obtained with M062x/6-311+g(2d, p). Conceptual Density Functional Theory is a chemical reactivity theory founded on Density-Functional-Theory-based concepts [43,44,45,46]. Within this theory there are response functions such as the electro-donating (ω−) and electro-accepting (ω+) powers, previously reported by Gázquez et al. [44,45]. The capacity to donate electrons (ω−) is defined as follows:

ω− = (3I + A)^2^/16 (I − A) (1)

whereas the propensity to accept electrons or ω+ is equal to:

ω+ = (I + 3A)^2^/16 (I − A) (2)

**Figure 2:**
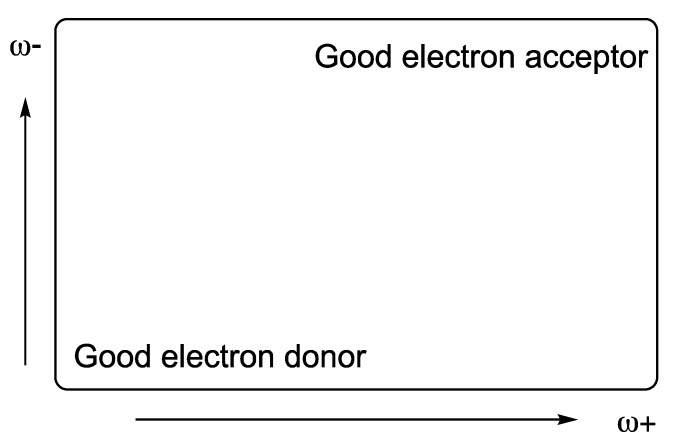
Electron Donor Acceptor Map (DAM).

I and A are the vertical ionization energy and vertical electron affinity, respectively. Low values of ω− indicate good electron-donor molecules. High values of ω+ are for good electron-acceptor molecules. These two quantities refer to charge transfers, not necessarily from one electron. These chemical descriptors have been used successfully in different chemical systems [30,47]. With these parameters it is possible to determine the Electron Donor Acceptor Map (DAM, see Figure 2) [30]. Systems located down to the left are considered good electron donors whilst those situated up to the right are good electron acceptors. 

I and A were obtained as follows:

A → A^+1^ + 1e    I = E (A^+1^) − E (A) (3)

A^−1^ → A + 1e    A = E (A) − E (A^−1^) (4)

These values were calculated with single-point calculations of the optimized geometries at the M062x/LANL2DZ level of theory. Solvent effects were included as single-point calculations with the optimized structures in vacuum, using the polarizable continuum model [42] with benzene as the solvent (all pigments are soluble in organic solvents). To evaluate the Hydrogen Atom Mechanism (HAT), the reaction energy (E_HAT_) was calculated as follows, considering the hydroxyl free radical (OH•):

X + OH• → X–H• + H_2_O   E_HAT_ = [E(X–H•) + E(H_2_O)] − [E(X) + E(OH•)] (5)

X represents PP, BV, or BR. Negative E_HAT_ values indicate energetically favorable HAT reactions. To compare with previous results for carotenoids [31], the dissociation energy (ΔE) of the H atom was calculated as expressed in the following equation:

X → X–H• + H              ΔE = E(X–H•) + E(H) − E(X)(6)

X–H• are the optimized and most stable dehydrogenated structures of PP, BV, or BR.

## 3. Results and Discussion

### 3.1. Free Radical Scavengers, Antioxidants, Antireductants, or Antiradicals

**Figure 3:**
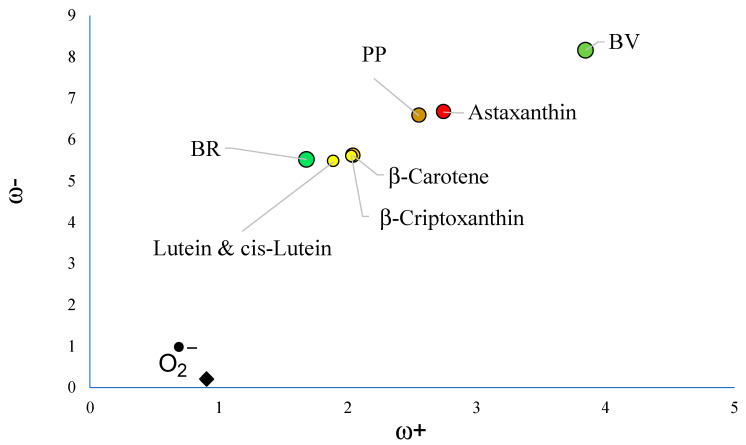
DAM of the molecules under study. Carotenoids (in yellow and red) are reported for comparison. The colors indicate the hue of the pigments. Values are in eV. Calculations were conducted in benzene to compare with previous results [34]. All molecules are in ground states (singlets).

To analyze the capacity to scavenge free radicals, the electron transfer capacity of the molecules under study were analyzed with the corresponding DAM (see Figure 3). Other pigments such as carotenoids are reported for comparison. As Figure 3 indicates, BV is the best electron acceptor among all molecules under study. It is better than astaxanthin and PP. Yellow carotenoids are better electron donors than BV and PP. Superoxide radical anion (O^2−^•) is also included and it can be seen that it is the best electron donor. Astaxanthin was previously reported as a good electron acceptor and for this reason a good free radical scavenger of the free radical O^2−^• [34]. 

To prevent oxidative stress, there are several mechanisms, with the electron transfer being one of the main ones. With this mechanism, the radical scavenging ability can be analyzed in two directions. Either the antioxidant donates an electron to the radical (the conventional mechanism), or the free radical scavenger takes an electron from the free radical. This last mechanism is specific to the superoxide radical anion (O^2−^•) [34]. It is worth remembering that the superoxide radical anion is related to several disorders associated with oxidative stress and is the main source of other reactive oxygen species. Therefore, capturing this free radical stops the production of free radicals and thus contributes to preventing oxidative stress. Free radical scavengers can be antioxidants (donate an electron) or antireductants (accept an electron) and we can also call them antiradicals. With this mechanism, it was possible to explain the extraordinary ability of red carotenoids such as astaxanthin to prevent oxidative stress, and this information has been used to analyze various experiments with carotenoids and also to explain observations of birds using carotenoids to indicate their “state of health” during the process of sexual selection [48,49,50,51,52,53,54,55,56]. This idea was also useful to study the physiological effects of heat stress on poultry health [57]. From the theoretical point of view, this mechanism and the DAM were proposed as a good tool to analyze the antioxidative nature of chemicals using computational quantum chemistry [58,59]. Concerning polymers, this idea was used to analyze natural antioxidants as stabilizers for polymers [60]. 

Carotenoids are found in feathers and not in eggshell as the pigments that we are investigating. They participate in the formation of colored feathers which is an important ornament of birds but not in eggshell color. As noted in the introduction, the main pigments responsible for the color of eggshells are PP (brown tones) and BV (greenish). These two pigments have an important role in reproductive success and one of the hypotheses is that these two pigments are capable of scavenging free radicals.

Considering the results of astaxanthin, it is possible to say that the best free radical scavenger of O^2−^• is BV. PP is a slightly worse electron acceptor than astaxanthin, and for comparison it could also be considered a good scavenger of superoxide radical anion. BR is not as good an electron acceptor as BV, but it is as good an electron donor as yellow carotenoids. Yellow carotenoids were reported as good free radicals considering the conventional mechanism, so it can be expected that BR as an antioxidant donates an electron to the free radical similar to yellow carotenoids. Altogether, these results indicate that PP, BV, and BR could be good scavengers of free radicals through the mechanism of electron transfer when they are in the ground state, with BV being the best antiradical.

Another important mechanism to scavenge free radicals is Hydrogen Atom Transfer (HAT) [31]. This mechanism considers the Hydrogen Atom Transfer from the antiradical to the free radical. This mechanism was previously reported for BR, due to the extended system of conjugated double bonds and the presence of two reactive hydrogen atoms. BR is an antioxidant via H donation to an incipient radical [28].

**Figure 4:**
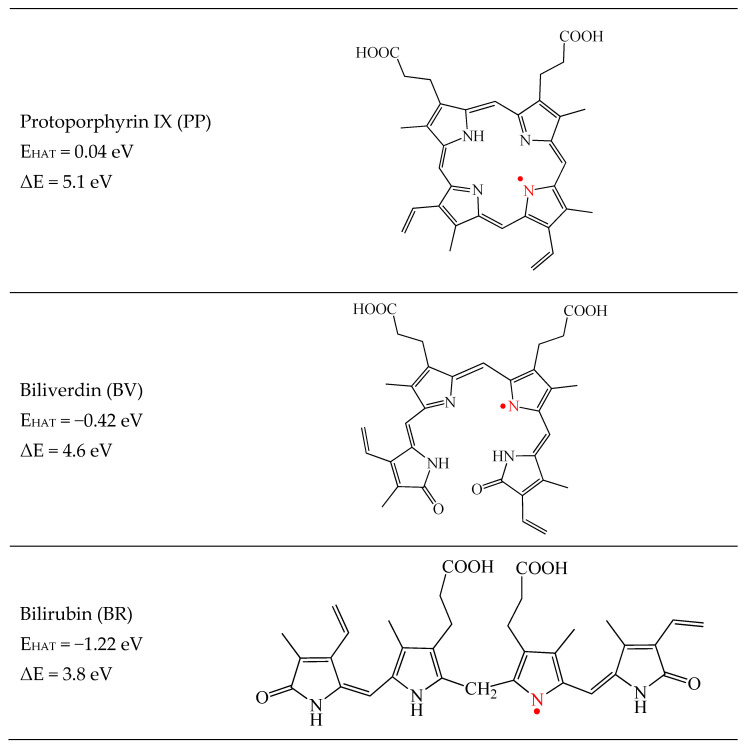
Most stable dehydrogenated optimized structures of the molecules under study. The reactive position is indicated in red. The dissociation energy (ΔE) is also reported for comparison with previous results for carotenoids [31]. Calculations are at the M062x/LANL2DZ level.

To investigate this mechanism, one hydrogen atom is removed from several positions of the molecules under study. The most stable dehydrogenated structures are reported in Figure 4. The reaction energy E_HAT_ is obtained with Equation (5) and it is also included. The dehydrogenated reaction of PP is not energetically feasible (E_HAT_ is small and positive). For BV, the value of E_HAT_ is −0.42 eV and for BR it is three times higher, −1.22 eV. Therefore, these two molecules could also scavenge free radicals considering this mechanism, with BR being a better antiradical than BV. This also corroborates the idea that it is a favorable mechanism of BR to capture free radicals.

To compare with previous results for carotenoids [31], the dissociation energies are included in Figure 4. The lower the ΔE value, the easier the H abstraction and the greater the potential role played by the molecule as an antiradical. The reported value for β-carotene and astaxanthin is the same (3.1 eV). The lowest value of the pigments analyzed in this investigation is 3.8 eV for BR. This means that carotenoids are better antiradicals than PP, BV, and BR, but these last two are also good free radical scavengers considering the HAT mechanism, whilst PP is not. Overall, our calculations show that PP, BV, and BR are free radical scavengers either transferring electrons or hydrogen atoms to free radicals.

### 3.2. Interaction with Ca^2+^

**Figure 5:**
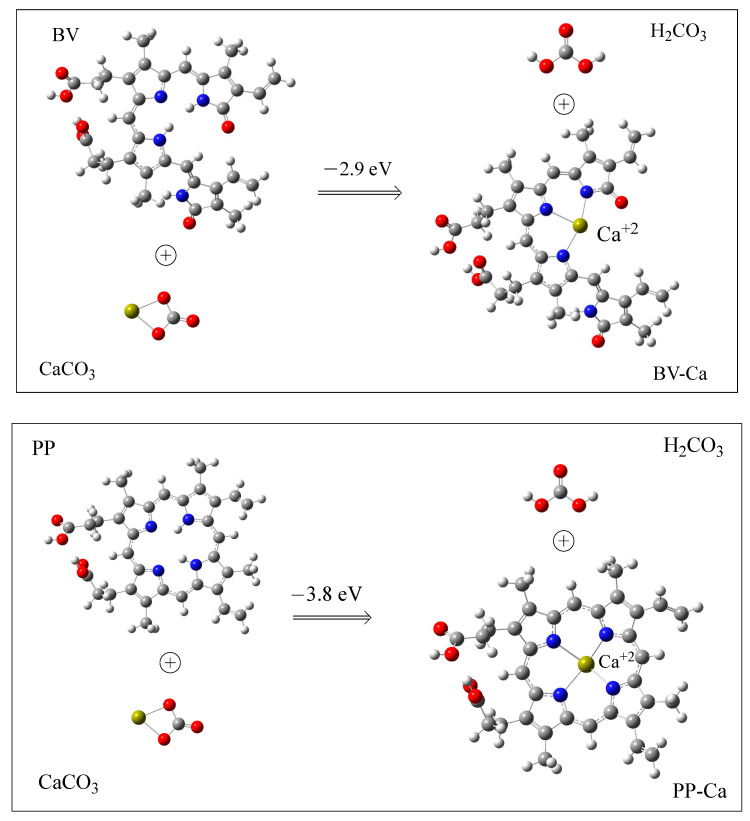
Most stable BV-Ca and PP-Ca structures. Grey indicates carbon atoms, red is for oxygen atoms, nitrogen atoms are in blue color, green indicates calcium and white is for hydrogen atoms Binding energies are also reported.

Our hypothesis to explain why PP is easily removed from recently laid eggs while this is not possible after some time is that PP forms a compound with Ca^2+^ and this incorporates the pigment into the calcium carbonate matrix. To analyze this option, we optimized the structures of PP and BV chelating Ca^2+^. Each chelated calcium displaces two hydrogen atoms from PP or BV, as was reported before for other metals [61]. BR with Ca^2+^ was not considered as this molecule is not an eggshell pigment and therefore it is not in contact with the calcium carbonate matrix. Figure 5 reports the optimized structures of BV-Ca and PP-Ca. Calcium cations are located in the middle of the molecules, as expected. BV forms three N–Ca bonds and PP forms four N–Ca bonds. Around calcium cation the structures are nearly planar. The bond distances are similar. For BV, there are two N–Ca bond distances that are equal to 2.4 Å and there is one that is 2.3 Å. The four Ca–N bond lengths of the PP systems are all the same and the values are equal to 2.3 Å. 

With this optimized structure, we obtained the formation energy, which is also reported in the figure. The values are −2.9 and −3.8 eV for BV and PP, respectively. This indicates that the reaction with PP is energetically more favorable than with BV, but BV can also form chelate compounds with Ca^2+^. This result with PP corroborates the hypothesis that the pigment could be incorporated into the calcium carbonate matrix and, therefore, could not be easily removed some time after laying. BV cannot be removed from the eggshell, even when they are newly laid, but our theoretical results indicate that the reaction to form the chelate is energetically feasible. The most important structural difference between these two molecules is the number of Ca–N bonds that are formed. With PP, there are four bonds and the calcium cation is in the macrocyclic ring of PP; whereas with BV, there are three bonds and they are in the open macrocyclic ring. This could influence the incorporation of BV-Ca into the calcium carbonate matrix, and this may explain why BV cannot be removed from the eggshell.

It is important to remember that we are not considering the structural organization of calcium carbonate in our calculations, and this could be important. In fact, there is strong evidence that the structural organization of a bird’s eggshell is genetically controlled, and different bird species have evolved eggshells with specific microstructural characteristics [6,13]. It is possible that green eggshells (with BV) have different microstructural characteristics than brown ones, and it could be that BV is incorporated into the calcium carbonate matrix during shell formation. If this were the case, then BV would never be removed from the eggshell, as it was part of the eggshell since its formation. At this point, more experiments are needed to investigate the interaction of BV with calcium.

**Figure 6:**
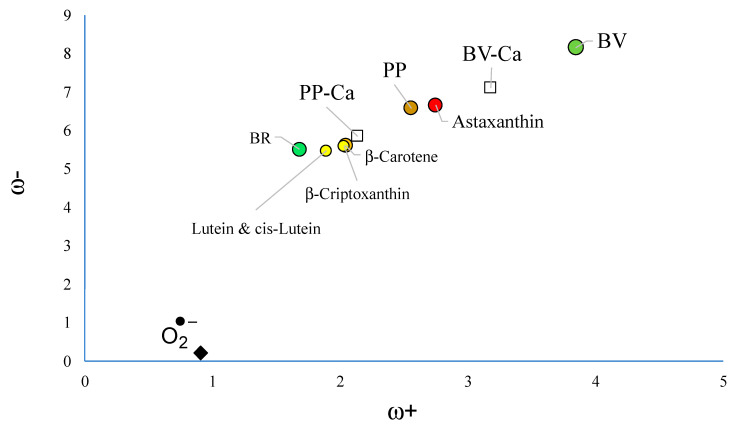
DAM of the molecules under study including compounds with Ca^2+^. Carotenoids (in yellow and red) are reported for comparison. The colors indicate the hue of the pigments. Values in eV.

Once we know that Ca^2+^ can form stable chelates with BV and PP, it is important to investigate whether the electron donor–acceptor properties of the chelated compounds are different from pigments without calcium. Figure 6 reports the DAM for all compounds under investigation. BV-Ca and PP-Ca are worse electron acceptors and better electron donors than BV and PP, respectively. BV-Ca is still a better electron acceptor than astaxanthin, so good antiradical properties can be expected even when chelated with Ca^2+^. PP-Ca is closer to yellow carotenoids than PP. It can be as good as these carotenoids in scavenging free radicals. These results are important when we considered the experimental results that are already reported [8,9,23]. The free radical scavenging capacity of these two pigments explains the correlation between egg pigmentation and female quality, and this is independent of the time after egg laying. Therefore, it is important that the presence of calcium in these molecules preserves their antiradical activity.

**Figure 7:**
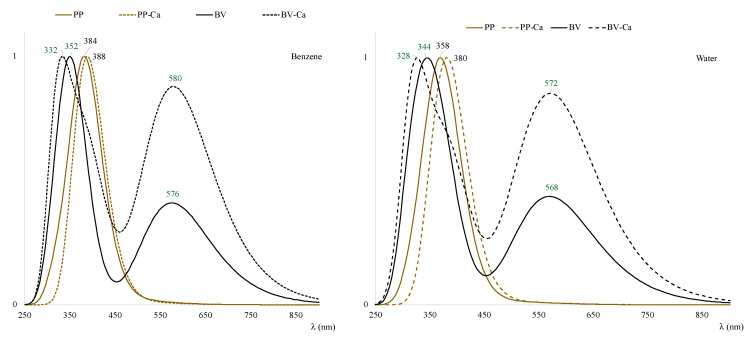
UV-visible spectra obtained with TDDFT using single-point calculations of the optimized geometries, at the same level of theory in benzene and water, to mimic nonpolar and polar environments.

The coloration of the eggshells is also important. Experimentally, no changes in the color of the eggshells are observed after some time. Brown eggshells are still brown and green ones remains green. To analyze the color in the presence of Ca^2+^, we obtained the UV-visible spectra in benzene and water to mimic nonpolar and polar environments (see Figure 7). As can be seen, the results are slightly different. In benzene, λ_max_ values are located at larger values than in water, but the results are similar.

In both environments, there are small changes in λ_max_, but it cannot be said that the presence of calcium modifies the color, in total agreement with the experimental observations. For BV, the experimental values are at low UV wavelengths (around 360 nm) and high green wavelengths (around 550 nm) [5]. This is in line with the results of Figure 7. The most important thing is that the color does not change due to the presence of calcium. 

### 3.3. Excited States and Photoactivation

To explain the antimicrobial defense through the photoactivation of PP, we calculated the triplet excited states of PP. We also obtain triplet states for BV for comparison. The excited states of both molecules were calculated with and without Ca^2+^. The excited state of molecular oxygen is the singlet oxygen, 1O_2_. This was also calculated since the mechanism of action for photoactivation is related to the production of 1O_2_ which then generates oxygen free radicals. The results are reported in Table 1. 

According to these values, 0.61 eV is necessary to obtain 1O_2_ from O_2_. PP and BV states will release 1.80 and 1.39 eV, respectively, when the excited triplets transfer energy to rich the most stable singlet states. Both can transfer energy from its triplet state to molecular-oxygen-producing singlet oxygen (1O_2_), which is the main mediator of photochemical cell damage. In this process, PP transfers more energy than BV. For the systems with Ca^2+^, the results are similar. This means that both pigments (with and without calcium) are able to produce singlet oxygen since the excitation energies are higher than the excitation energy of O_2_. Due to these results, both will trigger the production of oxygen free radicals. This is in line with the explanation of the idea that PP is pro-oxidant, and this contributes to antimicrobial defense. This mechanism of protection was not found for BV. However, it was stablished that BV has antimicrobial properties and may be able to inhibit viral replication in the eggshell due to its antiviral properties [25]. There is not a reported explanation concerning the antimicrobial and antiviral properties of BV. According to the theoretical results reported here, BV and BV-Ca could also be photoactivated by sunlight and transformed to excited triplet states. BV and BV-Ca triplets could transfer enough energy to molecular oxygen to produce 1O_2_. If this were the case, BV and BV-Ca might also be considered pro-oxidant molecules (in the excited state). More experiments are needed to verify these findings. 

## 4. Conclusions

The theoretical calculations indicate that PP, BV, and BR are free radical scavengers either transferring electrons or transferring hydrogen atoms to free radicals. These molecules are as good antiradicals as carotenoids. The interaction with calcium is energetically favorable for PP and BV. These two molecules form chelates with Ca^2+^. Calcium cations are located in the middle of the molecules. BV forms three N–Ca bonds and PP forms four N–Ca bonds. Around the calcium cation, the structures are nearly planar. The bond distances are similar. For BV, there are two N–M bond distances that are equal to 2.4 Å and there is one that is 2.3 Å. The four Ca–N bond lengths of the PP systems are all similar and equal to 2.3 Å.

As a result, pigments could be incorporated into the calcium carbonate matrix of the eggshell. This explains the experimental observations on the ease of removing PP just after egg laying and not when more time passes. BV is never easily removed. One explanation is that the calcium carbonate matrix of green eggshells could be different, so that BV can be immediately incorporated into the calcium carbonate matrix or even incorporated during eggshell formation. The most important structural difference between these two molecules is the number of Ca–N bonds that are formed. With PP, the calcium cation is in the macrocyclic ring of PP, whereas with BV it is in the open macrocyclic ring. This could influence the incorporation of BV-Ca into the calcium carbonate matrix, and this may explain why BV cannot be removed from the eggshell.

With both pigments, the presence of calcium in these molecules preserves their antiradical activity, and the UV-visible spectra are similar with and without calcium. 

The microbial defenses and the antiviral properties of these two pigments could be explained by the photoactivation of PP and BV by sunlight. PP and BV can produce singlet oxygen since their excitation energies are higher than the excitation energy of O_2_. Similar results are found for PP-Ca and BV-Ca. Due to these results, it might be possible that these molecules trigger the production of oxygen free radicals, and both can be considered pro-oxidants when in triplet excited states. 

## Figures and Tables

**Table 1:** Total energies (in au) for all the systems under study. The energy difference between triplets and singlets is also reported. Single-point calculations at the M062x/6-311+g(2d,p) level of theory, using optimized structures with M062x/LANL2DZ.

System	E (Triplet)	Singlet	ΔE (t-s)
PP	−1835.725452	−1835.791581	1.80 eV
BV	−1948.107976	−1948.158904	1.39 eV
PP-Ca	−2512.278101	−2512.345637	1.84 eV
BV-Ca	−2624.627534	−2624.677715	1.37 eV
O_2_	−150.313875	−150.291324	−0.61 eV

## Data Availability

All data are available under request to martina@unam.mx.

## Supplementary Materials

The following supporting information can be downloaded at: https://www.mdpi.com/article/10.3390/antiox12091662/s1. Cartesian coordinates and comparison between methods.
