# Supplementary material for: To Prevent Oxidative Stress, What about Protoporphyrin IX, Biliverdin, and Bilirubin?
Source: Antioxidants (Basel). 2023 Aug 23;12(9):1662. doi: 10.3390/antiox12091662 (PMC10525153; doi:10.3390/antiox12091662)
Supplement: Supplementary file 1 [file antioxidants-12-01662-s001.zip › antioxidants-2567549-supplementary.pdf]

To Prevent Oxidative Stress, What about Protoporphyrin IX, Biliverdin, and Bilirubin?

Ana Martínez [1], Isabel López-Rull [2] and Juan A. Fargallo [3]

Supplementary Material

Biliverdin (BV)

Results

M062x/LANL2DZ	Neutral	Cation	Anion	I	A	ω−	ω+
	−1947.332742	−1947.084837	−1947.409527	6.75	2.09	6.69	2.27
M062x/6-311+g(2d,p)							
	−1948.162669	−1947.920658	−1948.236540	6.59	2.01	6.47	2.17

Optimized Geometries

```
---------------------------------------------
 # opt freq LANL2DZ m062x
 ---------------------------------------------
                     Standard orientation:
 ---------------------------------------------------------------------
 Center     Atomic      Atomic             Coordinates (Angstroms)
 Number     Number       Type             X           Y           Z
 ---------------------------------------------------------------------
      1          8           0        4.432115   -2.974901    1.487050
      2          8           0        6.476830   -1.182099    1.785697
      3          8           0        4.782595   -2.147544   -0.599649
      4          8           0        7.429972    0.272309    0.313028
      5          8           0       -4.182138   -5.192601    1.849096
      6          8           0       -3.619298    1.931359   -0.807607
      7          7           0       -0.039156   -0.747762   -0.728932
      8          7           0        1.097347    1.784315   -0.481303
      9          7           0       -2.952758   -3.596190    0.633471
     10          7           0       -1.443398    2.758060   -0.516899
     11          6           0        1.197315   -2.557775   -1.249515
     12          6           0        1.254956   -1.164837   -0.997073
     13          6           0        3.434809    1.938978   -0.370924
     14          6           0        2.294327    1.064615   -0.644441
     15          6           0       -0.162289   -2.955844   -1.141222
     16          6           0        2.915492    3.172200   -0.044761
     17          6           0       -0.914497   -1.799186   -0.802065
     18          6           0        2.360709   -0.278668   -0.950480
     19          6           0        2.364822   -3.458746   -1.558656
     20          6           0        1.454027    3.038051   -0.138647
```

21	6	0	4.852769	1.440671	-0.334784
22	6	0	3.125741	-3.921662	-0.292227
23	6	0	-0.715486	-4.317055	-1.469420
24	6	0	-2.337094	-1.553980	-0.617274
25	6	0	5.086942	0.605470	0.943582
26	6	0	3.635822	4.429167	0.349378
27	6	0	0.494299	4.090677	0.121656
28	6	0	-3.222274	-2.373898	0.000255
29	6	0	-0.851393	3.930319	-0.034758
30	6	0	-4.680610	-2.131493	0.172533
31	6	0	-5.213059	-3.176552	0.873396
32	6	0	-1.957212	4.859944	0.279728
33	6	0	4.175398	-2.929618	0.155056
34	6	0	6.429466	-0.069541	0.933717
35	6	0	-4.113401	-4.135408	1.198264
36	6	0	-3.139557	4.221944	0.006655
37	6	0	-5.332343	-0.916455	-0.329380
38	6	0	-6.603275	-3.429078	1.359417
39	6	0	-2.823659	2.841239	-0.491522
40	6	0	-1.752904	6.231715	0.845139
41	6	0	-4.530333	4.633751	0.154299
42	6	0	-6.648583	-0.814956	-0.606981
43	6	0	-4.985260	5.891891	0.340446
44	1	0	3.339668	-0.709719	-1.142697
45	1	0	3.075701	-2.962302	-2.228683
46	1	0	1.996145	-4.346676	-2.082307
47	1	0	5.568567	2.267160	-0.369096
48	1	0	5.061680	0.808301	-1.205874
49	1	0	-0.225853	0.209328	-0.441480
50	1	0	-0.861960	1.988327	-0.835429
51	1	0	3.659311	-4.859043	-0.497595
52	1	0	2.446424	-4.112975	0.542310
53	1	0	-0.549467	-5.049478	-0.667937
54	1	0	-1.791108	-4.265812	-1.661211
55	1	0	-0.240054	-4.717925	-2.371095
56	1	0	-2.712336	-0.594982	-0.971040
57	1	0	5.038484	1.248992	1.832378
58	1	0	4.307252	-0.152147	1.062827
59	1	0	3.403769	5.252665	-0.336404
60	1	0	4.719741	4.282690	0.334751
61	1	0	3.357138	4.751820	1.359992
62	1	0	0.871124	5.044433	0.476221
63	1	0	-2.037469	-4.011389	0.740427
64	1	0	-4.701833	-0.041213	-0.483341
65	1	0	-6.576205	-4.157504	2.175053
66	1	0	-7.072382	-2.503280	1.711488
67	1	0	-7.238212	-3.845270	0.567823
68	1	0	-2.471820	6.425405	1.649132
69	1	0	-1.898531	7.001504	0.077905
70	1	0	-0.745585	6.350443	1.252694
71	1	0	-5.244230	3.814555	0.081039
72	1	0	5.155855	-2.342790	1.757226
73	1	0	7.382848	-1.559083	1.820125
74	1	0	-7.063047	0.122610	-0.963557
75	1	0	-7.331984	-1.653796	-0.517146
76	1	0	-6.048357	6.084380	0.441777

77	1	0	-4.330109	6.756344	0.370106

----

opt freq 6-311+g(2d,p) m062x

Standard orientation:

Center Number	Atomic Number	Atomic Type	Coordinates (Angstroms) X	Y	Z
1	8	0	4.304085	-3.014868	1.484164
2	8	0	6.450766	-1.036959	1.870579
3	8	0	4.789068	-2.170973	-0.521412
4	8	0	7.306733	0.203075	0.217606
5	8	0	-3.994585	-5.203711	1.931344
6	8	0	-3.707708	2.138104	-0.826735
7	7	0	-0.075162	-0.776753	-0.766472
8	7	0	1.053155	1.788979	-0.487025
9	7	0	-2.866203	-3.662046	0.628836
10	7	0	-1.525829	2.811304	-0.491232
11	6	0	1.201590	-2.529469	-1.286061
12	6	0	1.217190	-1.151108	-1.021804
13	6	0	3.377946	1.943077	-0.367710
14	6	0	2.236499	1.083159	-0.645922
15	6	0	-0.137339	-2.965141	-1.180930
16	6	0	2.861645	3.159728	-0.036086
17	6	0	-0.911140	-1.842315	-0.838855
18	6	0	2.306304	-0.248808	-0.950436
19	6	0	2.386756	-3.388015	-1.618546
20	6	0	1.408167	3.014067	-0.137392
21	6	0	4.791631	1.451128	-0.333107
22	6	0	3.127219	-3.911170	-0.375715
23	6	0	-0.651141	-4.333040	-1.517503
24	6	0	-2.333741	-1.638578	-0.641925
25	6	0	5.047016	0.640609	0.943446
26	6	0	3.571349	4.410589	0.373028
27	6	0	0.455647	4.065288	0.136257
28	6	0	-3.177004	-2.467598	0.001193
29	6	0	-0.884371	3.935755	-0.012106
30	6	0	-4.628160	-2.260079	0.196173
31	6	0	-5.112501	-3.289327	0.928431
32	6	0	-1.935168	4.911802	0.320756
33	6	0	4.145390	-2.932532	0.160921
34	6	0	6.377849	-0.054723	0.925597
35	6	0	-3.980661	-4.203070	1.253008
36	6	0	-3.136351	4.351784	0.033425
37	6	0	-5.328135	-1.085006	-0.322434
38	6	0	-6.477263	-3.560023	1.455635
39	6	0	-2.893892	2.969842	-0.486048
40	6	0	-1.653849	6.247409	0.922255
41	6	0	-4.495715	4.843775	0.184338
42	6	0	-6.607044	-1.084865	-0.703381
43	6	0	-4.866275	6.127356	0.208215
44	1	0	3.289009	-0.679132	-1.113676
45	1	0	3.100700	-2.842097	-2.242515
46	1	0	2.045098	-4.246839	-2.202902
47	1	0	5.500819	2.280749	-0.388599

48	1	0	4.996269	0.810788	-1.197833
49	1	0	-0.299178	0.154956	-0.443752
50	1	0	-1.012822	2.033795	-0.879229
51	1	0	3.702919	-4.808277	-0.634064
52	1	0	2.436091	-4.189254	0.423001
53	1	0	-0.449533	-5.069291	-0.729718
54	1	0	-1.729580	-4.312953	-1.688365
55	1	0	-0.178723	-4.707625	-2.430514
56	1	0	-2.741998	-0.698348	-1.002846
57	1	0	5.026747	1.289975	1.827185
58	1	0	4.265771	-0.109126	1.096350
59	1	0	3.313674	5.248289	-0.283719
60	1	0	4.655168	4.279631	0.332572
61	1	0	3.309424	4.703729	1.395749
62	1	0	0.838789	5.012834	0.497007
63	1	0	-1.932466	-4.008896	0.782095
64	1	0	-4.756583	-0.161864	-0.398374
65	1	0	-6.402112	-4.142867	2.376933
66	1	0	-7.014869	-2.627501	1.647884
67	1	0	-7.068027	-4.150073	0.746121
68	1	0	-2.476803	6.548609	1.575816
69	1	0	-1.542864	7.014786	0.148382
70	1	0	-0.733320	6.229744	1.509706
71	1	0	-5.251757	4.064406	0.249080
72	1	0	5.013533	-2.393053	1.735539
73	1	0	7.342743	-1.410153	1.809937
74	1	0	-7.072959	-0.176712	-1.068857
75	1	0	-7.211476	-1.985732	-0.688392
76	1	0	-5.908871	6.399911	0.325495
77	1	0	-4.156108	6.936856	0.081078
